# Anticancer Potential of Green Synthesized Silver Nanoparticles Using Extract of *Nepeta deflersiana* against Human Cervical Cancer Cells (HeLA)

**DOI:** 10.1155/2018/9390784

**Published:** 2018-11-01

**Authors:** Ebtesam S. Al-Sheddi, Nida N. Farshori, Mai M. Al-Oqail, Shaza M. Al-Massarani, Quaiser Saquib, Rizwan Wahab, Javed Musarrat, Abdulaziz A. Al-Khedhairy, Maqsood A. Siddiqui

**Affiliations:** ^1^Department of Pharmacognosy, College of Pharmacy, King Saud University, Riyadh, Saudi Arabia; ^2^Zoology Department, College of Science, King Saud University, P.O. Box 2455, Riyadh 11451, Saudi Arabia; ^3^Al-Jeraisy Chair for DNA Research, Zoology Department, College of Science, King Saud University, P.O. Box 2455, Riyadh 11451, Saudi Arabia

## Abstract

In this study, silver nanoparticles (AgNPs) were synthesized using aqueous extract of *Nepeta deflersiana* plant. The prepared AgNPs (ND-AgNPs) were examined by ultraviolet-visible spectroscopy, Fourier transform infrared (FTIR) spectroscopy, X-ray diffraction (XRD), transmission electron microscopy (TEM), scanning electron microscope (SEM), and energy dispersive spectroscopy (EDX). The results obtained from various characterizations revealed that average size of synthesized AgNPs was 33 nm and in face-centered-cubic structure. The anticancer potential of ND-AgNPs was investigated against human cervical cancer cells (HeLa). The cytotoxic response was assessed by 3-(4, 5-dimethylthiazol-2-yl)-2, 5-diphenyltetrazolium bromide (MTT), neutral red uptake (NRU) assays, and morphological changes. Further, the influence of cytotoxic concentrations of ND-AgNPs on oxidative stress markers, reactive oxygen species (ROS) generation, mitochondrial membrane potential (MMP), cell cycle arrest and apoptosis/necrosis was studied. The cytotoxic response observed was in a concentration-dependent manner. Furthermore, the results also showed a significant increase in ROS and lipid peroxidation (LPO), along with a decrease in MMP and glutathione (GSH) levels. The cell cycle analysis and apoptosis/necrosis assay data exhibited ND-AgNPs-induced SubG1 arrest and apoptotic/necrotic cell death. The biosynthesized AgNPs-induced cell death in HeLA cells suggested the anticancer potential of ND-AgNPs. Therefore, they may be used to treat the cervical cancer cells.

## 1. Introduction

Nobel metal nanoparticles have attracted the interest of scientific community due to their fascinating applications in the field of biology, material science, medicine, etc [1]. Silver nanoparticles specifically have gained attention due to their unusual physiochemical [2] (chemical stability and electrical conductivity) and biological activities such as antibacterial, antifungal, anti-inflammatory, antiviral, antiangiogenesis, anticancer, and antiplatelet activities [3–5]. In addition, silver nanoparticles have been used in clothing [6], room spray, laundry detergent, wall paint formulation [7, 8], sunscreens, and cosmetics [9]. Silver nanoparticles also inhibit HIV-1 virus from binding to the host cells *in vitro* [10]. Although a wide variety of metal nanoparticle preparation methods such as UV radiation, laser ablation, lithography, aerosol technologies, and photochemical reduction are available [11–13], the focus is shifting towards green synthesis of nanoparticles, using bacteria [14], yeast [15], fungi [16], and plants [17]. Green synthesis of nanoparticles reports to be clean, nontoxic, cost effective, and environmentally benign. Among the various biological methods available, the use of microbe-mediated synthesis has limited industrial use, as they require antiseptic conditions. On the contrary, the use of plant extract for the nanoparticles synthesis is valuable due to the ease of scale-up, less biohazardous nature, and avoiding the hideous procedure of maintaining the cell lines [18].

**Figure 1:**
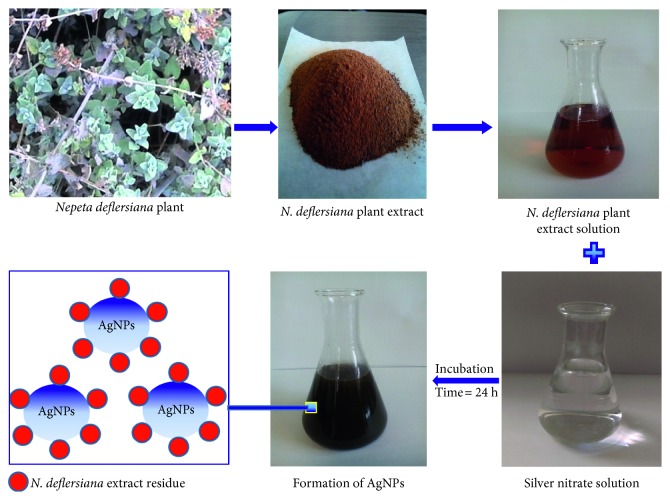
Schematic illustration of the green synthesis of silver nanoparticles (ND-AgNPs) using aqueous extract of the *Nepeta deflersiana* plant.

Cancer is a life threatening disease and leads the cases of deaths around the world [19]. According to the WHO, the annual cancer cases are to rise from 14 million in 2012 to 22 million in the next two decades [20]. Thus, the development of potent and effective antineoplastic drugs is one of the most persuaded goals. Among the various approaches, the exploitation of natural products is one of the most successful methods to identify novel hits and leads [21]. *Nepeta deflersiana* Schweinf. *(Labiatae)* is a medicinal plant growing in Saudi Arabia [22]. Traditionally *N. deflersiana* was used as a sedative; the leaf decoction was drunk with tea to release stomach and burn problems [23, 24]. The antimicrobial, anticancer, and antioxidant activities of *N. deflersiana* are documented [25]. Recently, we have reported the positive effects of *N. deflersiana* on human breast and lung cancer cell lines [26]. However, until the present, no published data are available on synthesis of nanoparticles using *N. deflersiana* plant. Herein, we report for the first time (i) the silver nanoparticles (ND-AgNPs) synthesis through a single-step silver ions reduction by *N. deflersiana* plant extract (Figure 1) and (ii) studied the anticancer activity of the biosynthesized silver nanoparticles against human cervical cancer (HeLa) cells.

## 2. Materials and Methods

### 2.1. Plant Material, Reagents, and Consumables

*Nepeta deflersiana (Lamiaceae)* plants were collected from Shaza Mountains, Saudi Arabia. The identity of the plant was confirmed by Dr. Jakob Thomas, KSU, and a voucher specimen (#15797) was deposited in the herbarium. Cell culture medium, antibiotics-antimycotic solution, trypsin, and FBS were procured from Invitrogen, USA. Plastic wares and other consumables were obtained from Nunc, Denmark. Other chemicals/reagents used in this study were purchased from Sigma, USA.

### 2.2. Preparation of Plant Extract

The aerial part of *N. deflersiana* was collected and washed several times with distilled water to remove dust and was dried under shade. The air-dried plant was cut into small pieces, macerated in distilled water, filtered under gravity, and the solvent evaporated under reduced pressure using a rotary evaporator. The dried extract was kept at 4°C (Figure 1).

### 2.3. Synthesis of Silver Nanoparticles

The aqueous extract of *N. deflersiana* (500 mg) was dissolved in 100 ml distilled water. Further 10 ml of the above extract was added to 90 ml of 0.1 M AgNO_3_ solution. After 24 h incubation, the solution turned dark brown, which indicates the formation of AgNPs. The solution was then transferred into a round bottom flask and was heated with continuous stirring at 90°C. After 15 min, the centrifugation was done at room temperature and a speed of 9000 rpm. The black powder obtained after washing thrice with distilled water was dried overnight in an oven at 80°C.

### 2.4. Characterization of Synthesized Silver Nanoparticles

The optical absorption of green synthesized silver nanoparticles was studied using FTIR (Shimadzu FT-IR Prestige 21) and UV-VIS (Shimadzu UV-VIS 2550, Japan) spectral analysis, respectively. Fourier transmission infrared (FTIR) spectra were recorded using KBr pellets in the range of 4000 to 400 cm^−1^. The crystalline nature of green synthesized AgNPs was confirmed by XRD pattern. The XRD data were recorded using PANalytical X'Pert X-ray diffractometer using Cu_K*α*_ (*λ* = 1.54056 Å). Morphology, size, and electron diffraction pattern were examined by SEM (JSM-7600F, Japan) and TEM (JEM-2100F, Japan) at a voltage 200 kV, respectively. EDX analysis was used to confirm the presence of elemental silver in green synthesized AgNPs.

### 2.5. Cytotoxicity by MTT Assay

Cytotoxicity of ND-AgNPs was examined by using MTT assay according to the method in [27]. In brief, HeLA cells obtained from American Type Culture Collection, USA, were plated in 96-well plates at a density of 1 x 10^4^ cells/well. Cells were exposed to 1–100 *μ*g/ml ND-AgNPs for 24 h. Following this, MTT was added in the wells, and plates were incubated for 4 h further. The reaction mixture was taken out and 200 *µ*l/well DMSO was added and mixed several times by pipetting up and down. The absorbance of plates was measured at 550 nm. The results were expressed as percentage of control.

### 2.6. Cytotoxicity by Neutral Red Uptake (NRU) Assay

Cytotoxicity by NRU assay was performed using the procedure [27]. Briefly, HeLA cells were treated with 1–100 *µ*g/ml ND-AgNPs for 24 h. Then, cells were washed with PBS twice and incubated further in 50 *µ*g/ml of neutral red containing medium for 3 h. The cells were washed off with a solution (1% CaCl_2_ and 0.5% formaldehyde). The dye was extracted in a mixture of 1% acetic acid and 50% ethanol. The plates were measured at 550 nm. The results were expressed as percentage of control.

### 2.7. Morphological Analysis

The changes in the morphology were observed under the microscope to determine the alterations induced by ND-AgNPs in HeLa cells treated with 1 *µ*g/ml to 100 *µ*g/ml of ND-AgNPs for 24 h. Images of the cells were grabbed at 20x by using the phase contrast inverted microscope (Olympus CKX 41, USA).

### 2.8. Glutathione (GSH) Level

The depletion in GSH level was measured following the protocol [28]. In brief, HeLA cells exposed to 5–25 *µ*g/ml ND-AgNPs for 24 h were centrifuged, and cellular protein was precipitated in 10% TCA (1 ml). Following this, supernatant was taken by centrifugation at 3000 rpm for 10 min. Then, 2 ml Tris buffer (0.4 M) with EDTA (0.02 M) and 0.01 M 5, 5'-dithionitrobenzoic acid (DTNB) were added in the supernatant. The absorbance was measured at 412 nm after incubating for 10 min at 37°C.

### 2.9. Lipid Peroxidation (LPO)

LPO in ND-AgNPs-exposed HeLA cells were measured following the method [28]. After respective treatment, cells were sonicated in chilled 1.15% potassium chloride solution. Following centrifugation, 1 ml of supernatant was added to 2 ml thiobarbituric acid solution (TCA (15%), TBA (0.7%), and 0.25 N HCl). The resulting solution was then boiled at 100°C for 15 min, and after the centrifugation for 10 min at 1000 × g, the absorbance was measured at 550 nm.

### 2.10. ROS Generation

The intracellular ROS generation was measured using 2, 7-dichlorodihydrofluorescein diacetate (DCFH-DA) dye [28]. In brief, HeLA cells were treated with different concentrations (10–50 *µ*g/ml) of ND-AgNPs for 24 h. The cells were then with DCFH-DA (5 *µ*M) at 37°C for 1 h. The cell pellet was collected in PBS (500 *μ*l) by centrifugation at 3000 rpm for 5 min. Then, the cells were analysed using flow cytometer.

### 2.11. Mitochondrial Membrane Potential (MMP)

The MMP level in HeLA cells was measured using the method defined by Zhang et al. [29]. In brief, HeLA cells were treated with 10 to 50 *μ*g/ml of ND-AgNPs for 24 h. Then, treated and untreated cells were incubated with rhodamine-123 (5 *μ*g/ml) for 1 h at 37°C in dark. Cells were washed twice, and finally, cell pellets were resuspended in PBS (500 *μ*l). MMP was measured by using flow cytometer.

### 2.12. Cell Cycle Analysis

ND-AgNPs-induced changes in cell cycle were measured using the protocol [30]. In brief, HeLa cells were exposed for 24 h at 10–50 *µ*g/ml ND-AgNPs. After the treatment, cells were fixed in chilled 70% ethanol for 1 h. Then, cells were washed twice by centrifugation, and cells were stained with propidium iodide for 60 min in dark. The stained cells were acquired by flow cytometer.

### 2.13. Apoptosis Assay

The apoptosis/necrosis induced by ND-AgNPs in HeLA cells were analysed using Annexin-V and 7-AAD Kit **(**Beckman Coulter**)** following the manufacturer's protocol. The amount of apoptosis/necrosis in the treated HeLa cells was analysed by flow cytometry following the protocol [31].

### 2.14. Statistical Analysis

Data were statistically analysed by ANOVA using the post hoc Dunnett's test. Value *p* < 0.05 was considered as a significant level between the exposed and control sets. The results are presented as mean ± standard deviation of three experiments.

## 3. Result and Discussion

### 3.1. Synthesis and Characterization of ND-AgNPs

**Figure 2:**
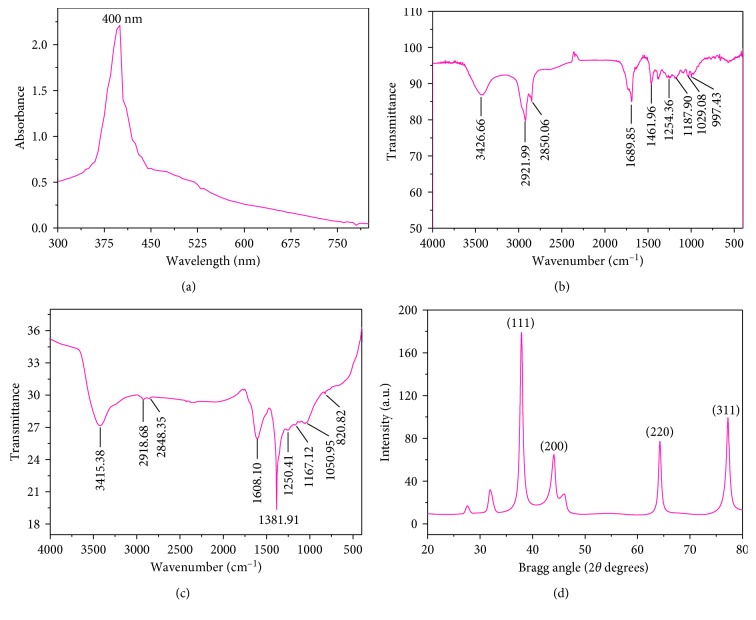
Characterization of green synthesized silver nanoparticles (ND-AgNPs) prepared using aqueous extract of the *Nepeta deflersiana* plant. (a) Ultraviolet-visible absorption spectra of synthesized silver nanoparticles (AgNPs). (b) Fourier-transform infrared spectra of *N. deflersiana* extract. (c) Fourier-transform infrared spectra of synthesized silver nanoparticles (ND-AgNPs). (d) X-ray powder diffraction pattern of synthesized silver nanoparticles (ND-AgNPs).

Plant extract of *N. deflersiana* was used for the synthesis of ND-AgNPs under facile conditions. The colorless silver nitrate solution (Figure 1) turned dark brown indicating the formation of silver nanoparticles (AgNPs). The occurrence of brown color can be attributed to the surface plasmons [32], arising from the collective oscillations of valance electrons in the electromagnetic field of incident radiation.  Figure 2(a) shows the UV-V is spectra of the synthesized AgNPs, giving the plasmon resonance at 400 nm. The characteristic *λ* max for AgNPs is in the range of 400–500 nm [33]. The position and shape of the surface plasmon absorption is dependent on the shape and size of particles formed, their interparticle distance, and the dielectric constant of the surrounding medium [34, 35]. Similar observations are reported earlier [32, 36]. FTIR measurements were carried out to identify the various functional groups in biomolecules responsible for the reduction of silver ions to AgNPs and capping/stabilization of AgNPs. The band intensities in different region of spectra for *N. deflersiana* extract (Figure 2(b)) and biosynthesized silver nanoparticles (Figure 2(c)) were analysed. The similarities between the two FTIR spectra, with some marginal shifts in peaks clearly indicate the plant extract is also acting as a capping agent. The *N. deflersiana* plant extract showed a number of peaks reflecting a complex nature of the plant extract. The shift in peaks at 3426 cm^−1^ corresponding to NH stretching of amide (II) band or C-O stretching or O-H stretching vibration implicated that their groups may be directly involved in the process of synthesis of AgNPs. Further, peak shifts from 1689 cm^−1^ to 1608 cm^−1^ indicated the possible involvement of C=O stretching or C-N bending in the amide group. Besides, the peak shifts from 1461 cm^−1^ to 1381 cm^−1^ suggest the involvement of C-H or O-H bending vibration of methyl, methylene, or alcoholic group in the reduction of Ag. Moreover, the observed peaks are more characteristic of flavonoids and terpenoids [37] that are present in the *Nepeta* species [25, 26]. It could be speculated that these secondary metabolites are responsible for the synthesis/stabilization of ND-AgNPs.

The crystalline structure of the green synthesized AgNPs was determined by XRD technique.  Figure 2(d) displays the XRD pattern of synthesized AgNPs. The Bragg reflection with 2*θ* values of 37.89, 44.23, 64.26, and 77.24 corresponding to (111), (200), (220), and (311) sets of lattice planes, respectively, is observed. These can be indexed to the face centered cubic (fcc) structure of the synthesized AgNPs. The crystalline size of the AgNPs was determined by using Debye–Scherrer equation [38]:

(1) $$\begin{array}{c} D=\frac{0.9\lambda }{\beta \cos\theta }, \end{array}$$

Where *D* is the grain size, *λ* is the wavelength of X-ray (1.54056 Å), and *β* is the full width at half maxima of the diffraction peak (in radians).

**Figure 3:**
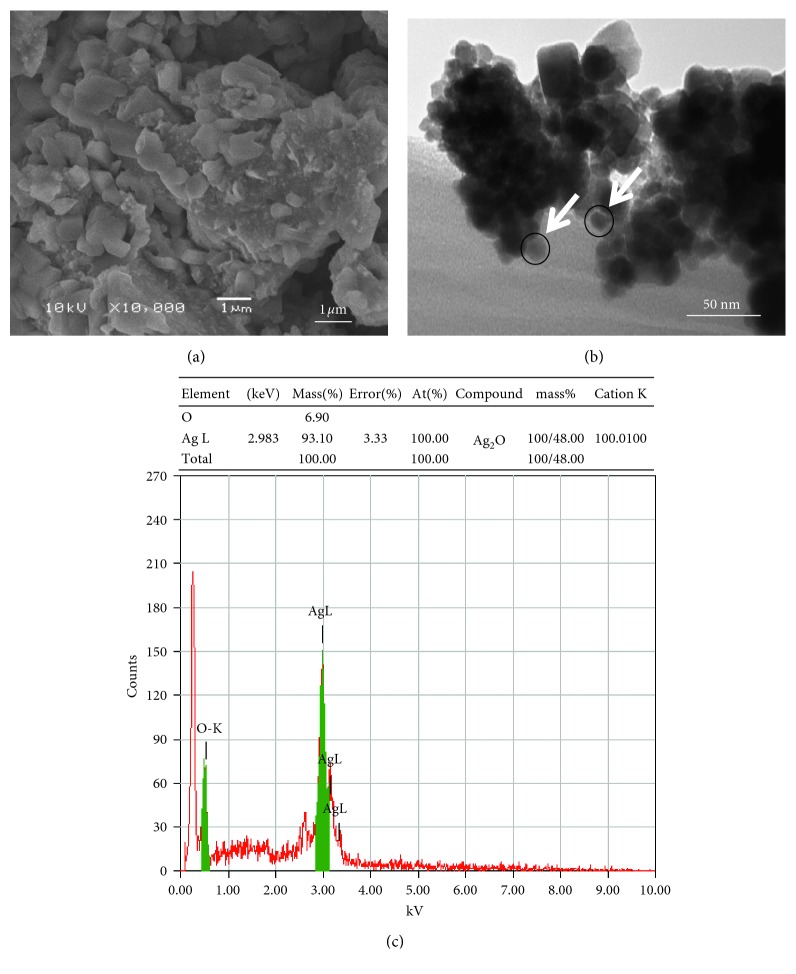
(a) SEM image of the green synthesized silver nanoparticles (AgNPs); (b) TEM image of green synthesized silver nanoparticles (ND-AgNPs) at 50 nm; (c) energy-dispersive X-ray spectrum of green synthesized silver nanoparticles (ND-AgNPs).

The average grain size determined by broadening of (111) reflection is estimated to be around 33 nm. Similar results have been reported earlier [39]. The absence of any reflection other than belonging to the silver lattice clearly indicates that the synthesized AgNPs lattice was unaffected by other molecules in the extract of plant. The scanning electron microscopy (SEM) and transmission electron microscopy (TEM) was employed to study the morphological and structural features of synthesized AgNPs. The SEM image (Figure 3(a)) shows that relatively spherical and uniform nanoparticles are formed. Some of the larger particles seen may be due to aggregation of nanoparticles induced by evaporation of solvent during sample preparation [40]. The TEM image (Figure 3(b)) revealed the nanoparticles formed have a narrow size distribution. The average size was about 33 nm, supporting the results of XRD further. Further, the energy-dispersion X-ray (EDX) spectroscopy study was employed to detect the existence of elemental silver.  Figure 3(c) shows the EDX image of *N. deflersiana* synthesized AgNPs. The results clearly indicate an intense signal at approximately 2.98 KeV corresponding to the presence of metallic silver nanocrystals, occurring due to surface plasmon resonance (SPR) [41]. The other intense signal at around 0.0–0.5 Kev represents the characteristic absorption for oxygen and carbon. This indicates the presence of *N. deflersiana* plant extract as a capping ligand on the surface of AgNPs.

### 3.2. Cytotoxicity Assessments of ND-AgNPs by MTT and NRU Assays

**Figure 4:**
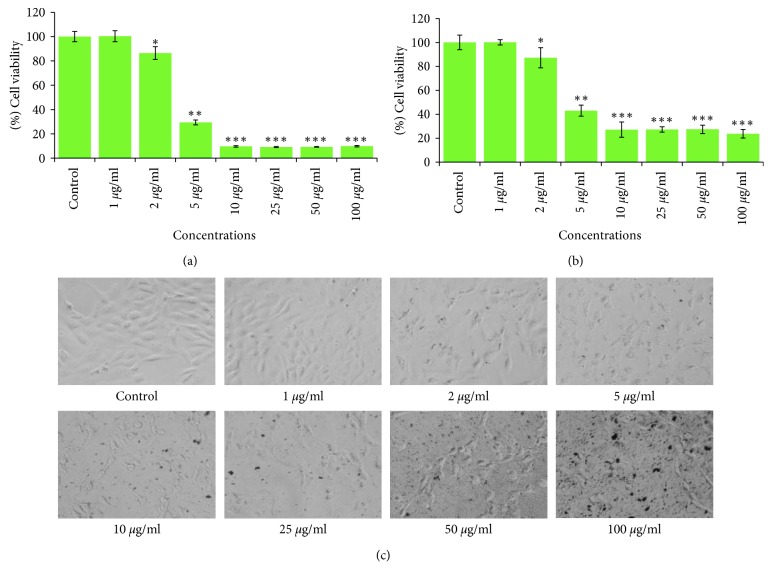
Cytotoxicity assessment in HeLA cells following the exposure of various concentrations of ND-AgNPs for 24 h: (a) MTT assay; (b) neutral red uptake assay. (c) Morphological changes. Images were taken using an inverted phase contrast microscope at 20x magnification. ^*∗*^*p* < 0.05,^*∗∗*^*p* < 0.01,^*∗∗∗*^*p* < 0.001 vs control.

The key results obtained by MTT and NRU assays in HeLA cells exposed to 1 *µ*g/ml to 100 *µ*g/ml for 24 h are summarized in Figures 4(a) and 4(b). The results exhibited a concentration dependent decrease in the viability of HeLA cells. The cell viability was recorded as 86% and 29% in ND-AgNPs at 2 *µ*g/ml and 5 *µ*g/ml concentrations, respectively; however, the maximum decrease in cell viability was measured as 9% each at 10, 25, 50, and 100 *µ*g/ml of ND-AgNPs (Figure 4(a)). Like MTT assay, a concentration-dependent decrease in cell viability of HeLA cells exposed to ND-AgNPs was also observed by NRU assay. The cell viability was recorded as 87% and 43% in ND-AgNPs at 2 *µ*g/ml and 5 *µ*g/ml concentrations, respectively; however, the maximum decrease in cell viability was measured as 23% at 100 *µ*g/ml of ND-AgNPs (Figure 4(b)). In this study, the cytotoxicity assessments were performed using two independent end points (MTT and NRU) assays [42]. The MTT, a colorimetric assay is based on the mitochondrial dehydrogenase enzyme of viable cells [43]; however, NRU assay is based on the lysosomal integrity of viable cells [44]. The cytotoxic responses of the ND-AgNPs, suggesting that biosynthesized AgNPs could contribute in search of alternative chemotherapeutic agent. Our results showed more than 50% of cell death even at 5 *µ*g/ml of ND-AgNPs. The cytotoxic effects induced by ND-AgNPs at lower concentrations could be due to the plant components attached to the AgNPs [45]. The results obtained from this study are also very well supported with various evidences for the cytotoxic effect of biosynthesized AgNPs using *Annona squamosa* leaf extract against the breast cancer MCF-7 cell line [46], *Piper longum* leaf extracts against Hep-2 cancer cell line [47], and *Morinda citrifolia* against HeLa cell lines [48] *in vitro*.

### 3.3. Morphological Analysis under the Microscope

The alterations observed in the morphology of HeLA cells treated with ND-AgNPs at 1–100 *µ*g/ml for 24 h are presented in Figure 4(c). There was no significant change observed in the morphology of control HeLA cells. The control cells appeared in normal shape and were attached to the surface. However, the HeLA cells exposed to ND-AgNPs lost their typical shape and cell adhesion capacity, shrinked, and decreased the cell density. These kind of changes have also been reported using plant synthesized AgNPs in different cancer cell lines [46], suggesting that the cytotoxic effect of synthesized AgNPs may be due to the antineoplastic nature and their capability *via* numerous molecular mechanism to induce cell death [45].

### 3.4. Glutathione Depletion and Lipid Peroxidation Level

**Figure 5:**
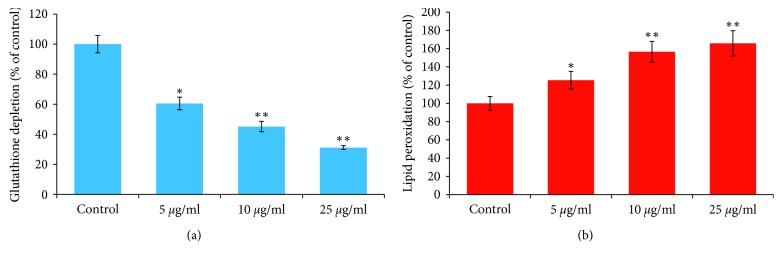
ND-AgNPs-induced oxidative stress in HeLA cells exposed for 24 h: (a) glutathione depletion; (b) lipid peroxidation. Results are expressed as the mean ± S.D. of three independent experiments. ^*∗*^*p* < 0.01,^*∗∗*^*p* < 0.001 vs control.

Figures 5(a) and 5(b) summarize the decrease in glutathione level and increase in the lipid peroxidation in HeLA cells exposed to ND-AgNPs at 5–25 *μ*g/ml concentrations for 24 h. The results indicate a concentration-dependent decrease in glutathione level. The depletion in the GSH was found to be 40%, 55%, and 69% at 5, 10, and 25 *μ*g/ml, respectively, as compared to control (Figure 5(a)). The effect of ND-AgNPs-induced lipid peroxidation in HeLA cells exposed for 24 h is shown in Figure 5(b). A concentration-dependent statistically significant increase in the LPO level was also observed in HeLA cells. The increase in LPO level was observed as 25%, 56%, and 65% at 5, 10, and 25 *μ*g/ml concentrations of ND-AgNPs, respectively (Figure 5(b)). Oxidative stress is known to be involved in the nanoparticles-induced cell death [49]. As observed in this study, the decrease in glutathione level and an increase in the level of lipid peroxidation suggest the role of oxidative stress in cell death in HeLA cell line exposed to ND-AgNPs. Our results are very well supported by previous report where a decrease in glutathione level and an increase in lipid peroxidation level have been observed due to the exposure of nanoparticles in various cell lines [49, 50].

### 3.5. Determination of Intracellular Reactive Oxygen Species (ROS)

**Figure 6:**
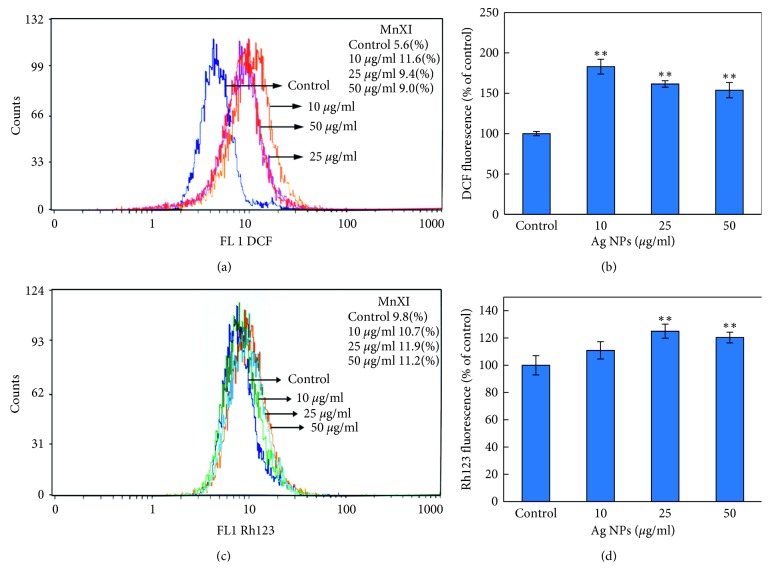
Flow cytometric analysis of intracellular ROS generation and mitochondrial membrane potential in HeLA cells exposed to ND-AgNPs for 24 h (a) Representative spectra of fluorescent DCF as a function of ND-AgNPs concentration. (b) Comparative analysis of the fluorescence enhancement of DCF with increasing concentrations of ND-AgNPs. (c) Representative spectra of fluorescence of Rh123 as a function of ND-AgNPs concentrations measured using a flow cytometer. (d) Comparative analysis of the fluorescence enhancement of Rh123 with increasing concentrations of ND-AgNPs. Each histogram represents mean ± S.D. values of DCF and Rh123 fluorescence obtained from three independent experiments. ^*∗∗*^*p* < 0.01 versus control.

The result obtained from ROS generation in HeLA cells exposed to ND-AgNPs for 24 h is shown in Figures 6(a) and 6(b). A statistically significant induction in ROS generation was measured in HeLA cells exposed to ND-AgNPs at 10, 25, and 50 *µ*g/ml concentrations. As shown in Figures 6(a) and 6(b), an increase of 207%, 167%, and 160% was observed in ROS generation at 5, 10, and 25 *μ*g/ml, respectively, as compared to untreated control. Nanoparticles are suggested to induce their toxicity through oxidative stress by generating reactive oxygen species (ROS) involved in a variety of different cellular processes ranging from apoptosis and necrosis to cell proliferation and carcinogenesis [51]. It have been reported that nanoparticles increase the ROS generation at cellular level. To investigate the potential role of ND-AgNPs in HeLA cell line, intracellular ROS generation was assessed by HDCF-DA dye using flow cytometer. An increase in the ROS level observed in this study established that AgNPs induced ROS generation, which leads to oxidative stress and cell death. Furthermore, consistent with previous reports that plant-synthesized AgNPs have capacity to induce ROS generation that can result in apoptotic cell death [52].

### 3.6. Mitochondrial Membrane Potential (MMP)

Figures 6(c) and 6(d) illustrate the change in the MMP level. HeLA cells were treated for 24 h at 10–25 *µ*g/ml of synthesized ND-AgNPs. A significant induction in MMP level was found in HeLA cells. The induction in MMP level was found to be 109%, 121%, and 114% at 5, 10, and 25 *μ*g/ml, respectively, compared to control set (Figures 6(c) and 6(d)). The results of this study suggested that the integrity of mitochondrial membrane might be involved in AgNPs-induced HeLa cell death. It is well documented that the ROS generation at high level can lead to cellular damage by resulting mitochondrial membrane damage, which can then induce toxicity [53, 54]. Based on cationic fluorescent probe Rh123 dye, the induction in MMP level indicated the role of reactive oxygen species generation and oxidative stress in the AgNPs-induced HeLA cell death due to free radicals generation [55].

### 3.7. Cell Cycle Analysis

**Figure 7:**
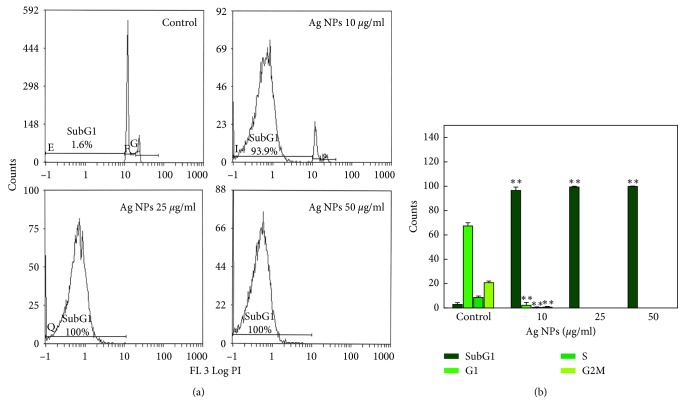
Cell cycle analysis in HeLA cells exposed to 10–50 *μ*g/ml concentrations of ND-AgNPs for 24 h. (a) Representative flow cytometric image exhibiting changes in the progression of cell cycle. SubG1 in each micrograph represents the percentage of cells in the SubG1 phase. (b) Each histogram represents the percentage of cells arrested in different phases of cell cycle. Results are expressed as the mean ± S.D. of three independent experiments. ^*∗∗*^*p* < 0.001 vs control.

The results of cell cycle analysis in HeLA cell lines exposed to ND-AgNPs at 10–50 *µ*g/ml for 24 h are represented in Figure 7. The flow cytometric measurement of propidium iodide-stained control and ND-AgNPs-treated HeLA cells showed an increase in apoptotic SubG1 peak. A significant increase in SubG1 arrest was observed at 50 *μ*g/ml concentrations of ND-AgNPs-treated HeLA cells (Figure 7). The increase in the SubG1 (apoptotic) population found in this study suggests that ND-AgNPs-treated HeLA cells were not able to go through G2 checkpoint; therefore, G2/M transition was found to be affected. The apoptosis induction due to the presence of SubG1 peak in the process of cell cycle suggests the role of early and late apoptotic/necrotic pathway [56, 57].

### 3.8. Apoptosis/Necrosis Assessment Using Annexin V-PE and 7-AAD

**Figure 8:**
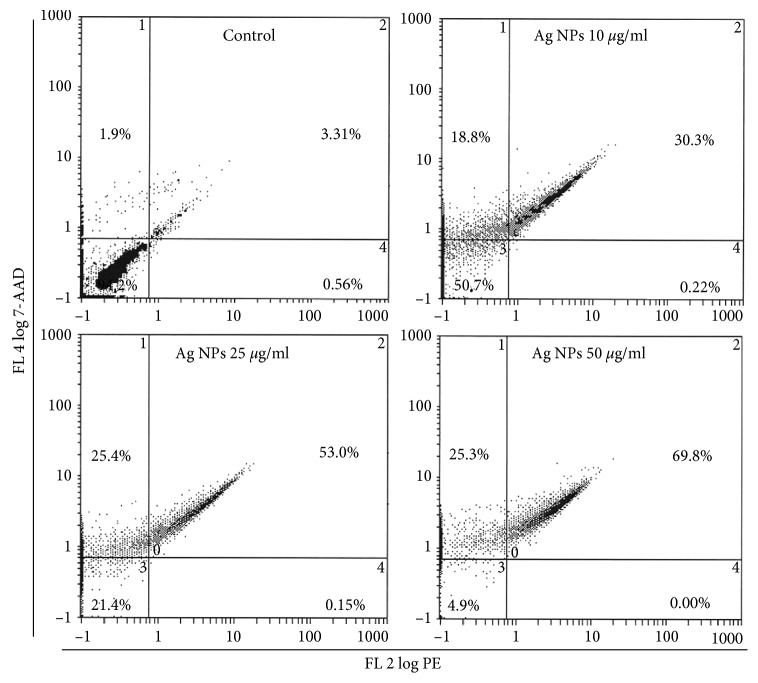
Annexin V-PE (phycoerythrin) and 7-AAD (7-amino actinomycin D assay. Bivariate flow cytometry analysis of HeLA cells treated with different concentrations of ND-Ag NPs. The scatter plots show early apoptotic, late apoptotic, and necrotic cells following 24 h treatment.

The results obtained from the induction of apoptosis/necrosis using flow cytometry are summarized in Figure 8. The flow cytometry data clearly showed that ND-AgNPs induced cell death in HeLA cells. Based on the Annexin V-PE/7-ADD staining, 94.2% of HeLA control cells were found alive with values of 0.56%, 3.31%, and 1.9% of cells, which are normal process for cells growing in cultures. The HeLA cells exposed to ND-AgNPs significantly increased the late apoptotic and necrotic cells as compared with untreated control cells. An increase in the percentage of apoptotic and necrotic cells was found with the values of 30.3–69.8% and 18.8–25.3% between 10 *µ*g/ml and 50 *µ*g/ml ND-AgNPs concentrations, respectively (Figure 8). Even at lower concentration, i.e., 10 *µ*g/ml, ND-AgNPs were found to induce apoptotic and necrotic cell death. It is well known that high amount of ROS generation could lead to apoptotic and necrotic cell death [58]. The excessive ROS generation has been linked with the substantial DNA damage and apoptosis/necrosis [59]. Our results are in well accordance with the recent reports that have shown apoptosis cell death due to the exposure of nanoparticles [60], including the exposure of plant-synthesized silver nanoparticles [52].

## 4. Conclusions

This investigation demonstrated the biosynthesis of silver nanoparticles (AgNPs) for the first time, via a single-step reduction of silver ions using *Nepeta deflersiana* plant and its anticancer potential against human cervical cancer (HeLa) cells. Our results showed that biosynthesized AgNPs (ND-AgNPs) induced a concentration-dependent cytotoxicity in HeLA cells. ND-AgNPs were also found to induce oxidative stress as observed by the increase in ROS and LPO level and the decrease in GSH level. The increase in the intracellular ROS generation was found eventually to trigger the development of mitochondrial membrane damage and cell cycle alterations. This study also showed that ND-AgNPs have the capacity of inducing apoptosis and necrosis cell death of HeLA cells through SubG1 cell cycle arrest. Thus, our findings suggest the anticancer potential of biosynthesized ND-AgNPs against human cervical cancer cells and could play an important role in the development of new therapeutic agent for the treatment of cancer.

## Data Availability

The data used to support the findings of this study are included within the article.
